# Emergence and spread of novel H5N8, H5N5 and H5N1 clade 2.3.4.4 highly pathogenic avian influenza in 2020

**DOI:** 10.1080/22221751.2021.1872355

**Published:** 2021-01-17

**Authors:** Nicola S. Lewis, Ashley C. Banyard, Elliot Whittard, Talgat Karibayev, Thamer Al Kafagi, Ilya Chvala, Alex Byrne, Saduakassova Meruyert (Akberovna), Jacqueline King, Timm Harder, Christian Grund, Steve Essen, Scott M. Reid, Adam Brouwer, Nikolay G. Zinyakov, Azimkhan Tegzhanov, Victor Irza, Anne Pohlmann, Martin Beer, Ron A. M. Fouchier, Sultanov Akhmetzhan (Akievich), Ian H. Brown

**Affiliations:** a Department of Virology, Animal and Plant Health Agency, OIE/FAO International Reference Laboratory for Avian Influenza, Swine Influenza and Newcastle Disease Virus, Surrey, UK; b Department of Pathobiology and Population Sciences, Royal Veterinary College, Addlestone, UK; c National Veterinary Reference Centre, Infectious Diseases Laboratory, The Committee for Veterinary Control and Supervision, Nur-Sultan City, Republic of Kazakhstan; d Veterinary Directorate, Baghdad, Iraq; e National Reference Laboratory for Avian Influenza and Newcastle Disease, Federal Centre for Animal Health (FGBI “ARRIAH”), Yur'evets Vladimir, Russia; f Department of Virology, The Kazakh Scientific Research Veterinary Institute (KazSRVI), Non-Commercial JSC “National Agrarian Science and Educational Centre”, Almaty, Republic of Kazakhstan; g Erasmus MC Department of Viroscience, Rotterdam, Netherlands; h Institute of Diagnostic Virology, Friedrich-Loeffler-Institut, Federal Research Institute of Animal Health, Greifswald-Insel Riems, Germany

**Keywords:** Influenza, emergence, HPAI, Eurasia, avian

## Abstract

Analyses of HPAI H5 viruses from poultry outbreaks across a wide Eurasian region since July 2020 including the Russian Federation, Republics of Iraq and Kazakhstan, and recent detections in migratory waterfowl in the Netherlands, revealed undetected maintenance of H5N8, likely in galliform poultry since 2017/18 and both H5N5 and H5N1. All viruses belong to A/H5 clade 2.3.4.4b with closely related HA genes. Heterogeneity in Eurasian H5Nx HPAI emerging variants threatens poultry production, food security and veterinary public health.

## The emergence of H5N8 HPAIV across Eurasia

**Figure 1.**
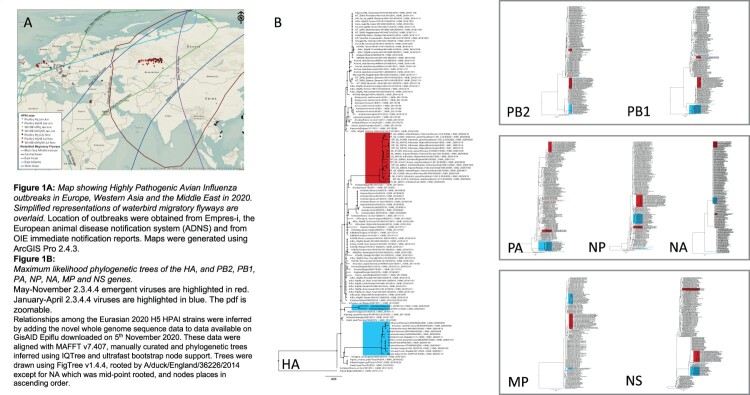
(A) Map showing highly pathogenic avian influenza outbreaks in Europe, Western Asia and the Middle East in 2020. Simplified representations of waterbird migratory flyways are overlaid. Location of outbreaks was obtained from Empres-i, the European animal disease notification system (ADNS) and from OIE immediate notification reports. Maps were generated using ArcGIS Pro 2.4.3. (B) Maximum likelihood phylogenetic trees of the HA, and PB2, PB1, PA, NP, NA, MP and NS genes. May–November 2.3.4.4 emergent viruses are highlighted in red. January–April 2.3.4.4 viruses are highlighted in blue. The pdf is zoomable. Relationships among the Eurasian 2020 H5 HPAI strains were inferred by adding the novel whole genome sequence data to data available on GisAID Epiflu downloaded on 5th November 2020. These data were aligned with MAFFT v7.407, manually curated and phylogenetic trees inferred using IQTree and ultrafast bootstrap node support. Trees were drawn using FigTree v1.4.4, rooted by A/duck/England/36226/2014 except for NA which was mid-point rooted, and nodes places in ascending order.

Highly pathogenic avian influenza (HPAI) viruses continue to threaten poultry production and food security throughout Eurasia, the Middle East and Africa [1]. Here we characterize the emerging events in Eurasia and assess evolution, diffusion and risk mitigation. Since 2008, the H5Nx clades have steadily evolved to constitute a genetically and antigenically broad series of isolates (assessed in [2]). Prior to the detection of H5N8 viruses in poultry in the Republic of Iraq in May 2020, in Russia in July and August 2020 and in Kazakhstan during September 2020, small H5N8 outbreaks had been reported in early 2020 across the European poultry sector (Figure 1(A)). The HA genes of European clade 2.3.4.4b H5N8 viruses detected during the first six months of 2020 clustered into two distinct groups – one within Bulgaria with genetic relationships to previously detected viruses in the country since 2018, and one monophyletic clade of viruses from early 2020 detected in Hungary, the Czech Republic, Germany and Poland. The phylogenetic patterns within Europe in early 2020 suggested that some H5N8 viruses were being maintained in European poultry whilst additional new variants from unknown sources were being sporadically introduced into other European countries e.g. Hungary, Germany, the Czech Republic and Poland with detection in wild and domestic birds (Figure 1(B)).

In May 2020, the Republic of Iraq reported H5N8 in poultry, having not detected outbreaks for over a year. Whole genome sequence data demonstrated that a new H5 2.3.4.4b variant had emerged, with a complete gene cassette indicating the closest genetic relatives as being those from the Eurasian 2.3.4.4b H5 outbreaks in 2017/18. The long branch lengths for all segments to these closest relatives suggested that the virus had been circulating undetected for the intervening period and that this circulation possibly occurred in galliform poultry, as there was no evidence of reassortment (Figure 1(B)).

Following outbreaks in Iraq, detections of H5N8 in the Chelyabinskaya Oblast (Chelyabinsk), southern central Russia were reported in late July 2020 and impacted on backyard chickens, geese and ducks. Interaction with wild birds was stated as the most likely incursion route [3]. A total of 11 cases were described in the period from August to September in Tyumen, Omsk and Kurgan.

Concurrent with H5 detections in Russia were outbreaks in neighbouring Kazakhstan, in backyard poultry, across the north-central region of the country, including Kostanay, Akmola, and Pavlodar. Culling of flocks was undertaken alongside implementation of surveillance zones and vaccination. Samples submitted by the National Veterinary Reference Centre, Kazakhstan to the Animal and Plant Health Agency, UK on FTA cards were triaged diagnostically for viral nucleic acid. All samples were positive for H5N8 nucleic acid by subtype-specific real-time RT-PCR [4,5]. Genetic analyses of the whole genomes from the Kazakhstan outbreaks demonstrated that for all segments they were highly similar to the H5N8 virus from Iraq as evidenced by relatively short branch lengths within this Iraqi-like cluster linked by a long branch to ancestral strains (Figure 1(B)).

H5 HPAI was detected in nine Eurasian Wigeons (*Mareca Penelope*) in the Netherlands that were caught on October 16, and whole genome sequences were generated for four of these and deposited onto GISAID (Figure 1(B)). The H5N8 virus detected in one Wigeon (A_Eurasian_Wigeon_Netherlands_7_2020_AH5N8_2020_10_16) was “Iraqi-like” for all gene segments. However, the H5N1 viruses (n=3) had an HA and MP gene segment which were “Iraqi-like” but there was evidence for reassortment with Eurasian avian lineage LPAI genes in all other gene segments. Closest relatives for these gene reassortants ranged in location from across Eurasia and North Africa and in time from 2010 to 2019, suggesting under-surveillance of the genetic diversity of avian influenza viruses in both wild and domestic birds and that attempting to estimate the time to most recent common ancestor for this emergence would likely be confounded by such under-surveillance.

H5 HPAI was detected in a Common buzzard (*Buteo buteo*) on 29 October and a Barnacle Goose (B*ranta leucopsis)* on 30 October in Germany, and whole genome sequences were generated and deposited onto GISAID. The H5N8 virus detected in the barnacle goose was “Iraqi-like” for all gene segments. However, the H5N5 virus detected in the buzzard was “Iraqi-like” for all genes except PA, where it clustered with Russian Federation LPAI H3N1 viruses from 2018. The N5 NA from the buzzard clustered with the H5N5 from the Russian Federation. None of the gene segments from the latest German viruses, detected between 16th January and 27th March 2020, clustered with earlier 2020 outbreak strains from Europe.

On 1 November H5 HPAI was detected in a chicken in the United Kingdom. Genetic analyses showed that this H5N8 virus was entirely “Iraqi-like”. To date, there have been no wild bird detections in the UK.

Across these detections, the HA cleavage site was poly-basic in nature for all analysed viruses (PLREKRRKRGLF), therefore satisfying international criteria for a HPAIV. Analysis of HA data enabled typing of the HA clade and all samples from Iraq, the Republic of Kazakhstan, and the Netherlands were classified as HA clade 2.3.4.4b utilizing the proposed update to the unified nomenclature for HPAI A(H5) viruses [6]. Comparisons with putative vaccine strains have shown genetic heterogeneity in the HA, and the need to subtype match the NA has not been assessed but might impact vaccine performance.

## Evaluation and risk mitigation

The emergence of these clade 2.3.4.4b H5 viruses in Eurasia has significant implications for the poultry sector across Europe, Central Asia, The Middle East and Africa during winter 2020–2021 [7]. An alert has already been raised for increased vigilance across northern and eastern Europe based on historic outbreaks in Russia and subsequent spread of disease in both 2005 and 2016 [8,9]. The heterogeneity already detected in these emergent viruses poses significant challenges in assessing host tropism, pathogenesis, routes of transmission and disease dispersal and above all defining efficacious risk mitigation strategies. Enhanced surveillance in both wild and domestic birds is warranted and should also focus on both domestic galliform and anseriform production systems. This should include assessment of genetic diversity of all Influenza A viruses to enable more accurate determinations of virus evolution including through reassortment. If vaccination is considered it is imperative to strain match as closely as possible, given the substantial amino acid mismatches already evident at the HA-level.

Pathways for transmission of these variants are unclear at this stage. The short branch lengths within the “Iraqi-like” cluster might indicate a role for poultry-mediated or indirect transmission at least initially in Central Asia. The H5N8 virus and the H5N1 virus reassortant clearly infected wild birds migrating to Europe as evidenced by the Wigeons in the Netherlands, but whether the H5N1 or H5N8 virus would be better maintained in wild anseriformes and therefore potentially diffuse more readily is uncertain. In 2014/15 the H5N8 virus was diffused by wild birds but also showed very little evidence of reassortment [10]. Conversely in 2016/17 there was substantive reassortment in wild birds whilst in early 2020 in Europe it was proposed that newly detected H5N8 viruses were derived from reassortment of sub-Saharan Africa viruses with those of LPAI of Eurasia origin [10,11]. Here we have co-circulation of at least three 2.3.4.4b H5 variants. Key to mitigating the risk to poultry will be understanding ongoing evolution, identifying where these viruses emerged from, mitigating endemic disease in poultry to avoid these production systems potentially acting as a future source for emerging variants and characterizing host tropisms and the role of wild birds in spreading disease.
